# Cell culture model predicts human disease: Altered expression of junction proteins and matrix metalloproteinases in cervical dysplasia

**DOI:** 10.1186/1472-6890-12-9

**Published:** 2012-08-03

**Authors:** Niina Kivi, Mikko Rönty, Jussi Tarkkanen, Petri Auvinen, Eeva Auvinen

**Affiliations:** 1 Haartman Institute, Department of Virology, University of Helsinki, POB 21 (Haartmaninkatu 3), FIN-00014, Helsinki, Finland; 2 Department of Pathology, Helsinki University Hospital Laboratory, Helsinki, Finland; 3 DNA Sequencing and Genomics Laboratory, Institute of Biotechnology, University of Helsinki, Helsinki, Finland; 4 Department of Virology and Immunology, Helsinki University Hospital Laboratory, Helsinki, Finland

**Keywords:** Cadherin, Catenin, CIN, Cytokeratin, E5, HPV, Microarray, MMP

## Abstract

**Background:**

Cervical cancer is necessarily caused by human papillomaviruses, which encode three oncogenes manifesting their functions by interfering with a number of cellular proteins and pathways: the E5, E6, and E7 proteins. We have earlier found in our microarray studies that the E5 oncogene crucially affects the expression of cellular genes involved in adhesion and motility of epithelial cells.

**Methods:**

In order to biologically validate our previous experimental findings we performed immunohistochemical staining of a representative set of tissue samples from different grades of high-risk human papillomavirus associated cervical disease as well as normal squamous and columnar cervical epithelium. Three-dimensional collagen raft cultures established from E5-expressing and control epithelial cells were also examined. The expression of p16, matrix metalloproteinase (MMP) -7, MMP-16, cytokeratin (CK) 8/18, laminin, E-cadherin and beta-catenin was studied.

**Results:**

In agreement with our previous microarray studies, we found intense staining for E-cadherin and beta-catenin in adherens junctions even in high-grade cervical lesions. Staining for MMP-16 was increased in severe disease as well. No significant change in staining for MMP-7 and cytokeratin 8/18 along with the grade of cervical squamous epithelial disease was observed.

**Conclusions:**

Here we have confirmed, using tissue material from human papillomavirus associated lesions, some of the cellular gene expression modifications that we earlier reported in an experimental system studying specifically the E5 oncogene of papillomaviruses. These findings were partially surprising in the context of cervical carcinogenesis and emphasize that the complexity of carcinogenesis is not yet fully understood. Microarray approaches provide a wide overwiev of gene expression in experimental settings, which may yield biologically valid biomarkers for disease diagnostics, prognosis, and follow-up.

## Background

Cervical cancer is necessarily caused by human papillomaviruses (HPV) [1]. Cancer-associated high-risk papillomavirus types (hrHPV) confer their oncogenic functions with the help of viral E5, E6 and E7 oncogenes. The immortalizing and transforming properties of the E6 and E7 oncogenes have been well established in experimental systems, and these properties are in agreement with the epidemiological data on the association of different virus types with human cancer [2]. The functions of the E5 oncogene are not as thoroughly understood, although it is known to stimulate epidermal growth factor receptor (EGFR) signaling, cell proliferation and immortalization of keratinocytes [3-7]. In a transgenic mouse model E5 was recently suggested to have a crucial role in cervical carcinogenesis [8].

In normal squamous epithelium, E-cadherin–beta-catenin complexes are important in maintaining the integrity of adherens junctions between two adjacent cells, as well as the barrier capacity of the epithelium [9]. Carcinogenesis is understood to involve breakdown of adherens junctions, which is seen in reduced expression or absence of these proteins in intercellular junctions [9]. We have previously explored the effects of the HPV 16 E5 oncogene on the expression of cellular genes and microRNAs in two microarray studies using stable E5-expressing HaCaT keratinocytes and control cells in order to understand the complexity of the E5 functions [10,11]. Genes involved in cell motility, cell adhesion and extracellular matrix were overrepresented among the genes whose expression was significantly altered due to E5 expression [10,11]. In validation experiments we showed upregulated expression of E-cadherin and beta-catenin proteins, important components of adherens junctions in epithelial cells, in monolayer as well as in three-dimensional collagen raft cultures of E5 expressing cells [11]. The expression of N-cadherin was also found to be upregulated. In agreement with these findings, we also observed downregulation of miR-324-5p, a cellular microRNA predicted to target both E-cadherin and N-cadherin expression [11].

Degradation of the extracellular matrix is an essential event in carcinogenesis and it requires the activity of matrix metalloproteinases [12]. Somewhat surprisingly, in our previous microarray experiments we found that expression of HPV 16 E5 downregulates the expression of matrix metalloproteinase (MMP) -7, MMP-12 and MMP-16 mRNA, although protein levels were similar in E5 expressing and in control cells [10,11]. Further, enhanced signaling downstream of fibronectin was suggested by the observed upregulation and increased activation of paxillin, and increased cell motility was confirmed in live cell imaging of wounded monolayer cell cultures [10]. Altogether, the previously reported effects of the HPV 16 E5 protein on cellular gene expression seem to favor cell proliferation and tumorigenesis and repress epithelial differentiation, although these findings are not completely unequivocal [10,11].

The E6 and E7 proteins of hrHPV have a number of functions associated with key carcinogenic events in epithelial cells. However, the plethora of activities ascribed to the E5 protein awaits biological validation. Here we sought out to study whether the alterations seen at gene expression level in tissue culture cells would be manifested in cervical HPV associated disease. We were also interested in the possibility of finding feasible biomarkers for the diagnosis, prognosis and follow-up of cervical disease among the genes and pathways affected by the E5 protein. Our results suggest that experimental approaches to the functions of a single oncogenic protein may prove biologically relevant in the context of a tumor virus, which encodes several proteins interfering with key oncogenic functions. We were able to confirm several of the original microarray findings, even surprising ones, in human cervical disease, which is the result of concerted action of viral proteins in the epithelium. These observations may contribute to our understanding of HPV pathogenesis and cervical carcinogenesis, and provide putative biomarkers for future patient care.

## Methods

### Patients

A total of 50 archival samples fixed in 10 % formaline and embedded in paraffin were collected from the archives of the Department of Pathology, Helsinki University Central Hospital. The material comprised samples from 4 condyloma, 15 cervical intraepithelial neoplasia grade 1 (CIN1), 27 CIN2, 2 CIN3, and 2 adenocarcinoma in situ (AIS) cases. Four samples from normal squamous epithelium and two samples from normal columnar epithelium with endocervical glands were studied as well. The use of archival samples was approved by the Coordinating Ethical Committee of the Helsinki University Central Hospital.

### Preparation of collagen raft cultures

Collagen raft cultures were prepared using a HaCaT cell line with stable HPV 16 E5 gene expression (HaCaT-E5) and a control HaCaT cell line without the E5 gene (HaCaT-pMSG) [13] to produce a three-dimensional tissue culture mimicking differentiating layered epithelium [14]. Similar cultures were produced using human keratinocytes expressing HPV16 E6 or E7 protein. After 21 days of culture, the epithelial cell rafts were first fixed overnight in 0.1 % glutaraldehyde/1 % formaldehyde/2 % Bacto agar containing salts and buffer, further fixed overnight in 10 % formaline, and embedded in paraffin.

### Immunohistochemical staining

For immunohistochemical staining, 4–5 μm sections were prepared and immunostainings were performed using the automatic Ventana Discovery tissue staining instrument (Ventana Medical Systems, Tucson, AZ). Antibodies for the individual cellular proteins are described in Table 1. Ventana DAB Map kit was used for detection, and the sections were counterstained with hematoxylin and postcounterstained with Bluing Reagent (Ventana Medical Systems). Finally, the slides were rinsed and dehydrated before mounting with EuKitt Mounting Medium (Fluka, Sigma-Aldrich Inc., Saint Louis, MO). Thickness and intensity of each staining were evaluated and given a value 0–3. The average staining thickness and intensity rankings for each morphological class as well as for normal tissues are given in Table 2.

**Table 1:** Primary antibodies used in tissue staining experiments

**Antibody**	**Identification**	**Dilution**	**Source**
p16INK4a	E6H4	undiluted	mtm laboratories AG, Heidelberg, Germany
MMP-7	Clone ID2	1:30	Calbiochem, Merck Chemicals Ltd., Nottingham, UK
MMP-12	rabbit polyclonal	1:50	Sigma-Aldrich Inc., Saint Louis, MO
MMP-16	rabbit polyclonal	1:50	Sigma-Aldrich Inc., Saint Louis, MO
CK8/18	Clone 5D3	1:50	Novocastra Lab’s Ltd., Vision Biosystems, Newcastle, UK
Laminin	rabbit polyclonal	1:350	DakoCytomation, Glostrup, Denmark
EDA-Fibronectin	Clone 52DH1	1:30	Dr Ismo Virtanen [15]
E-cadherin	Clone 36	1:200	BD Transduction Laboratories, Franklin Lakes, NJ
N-cadherin	Clone GC-4	1:30	Sigma-Aldrich Inc., Saint Louis, MO
Beta-catenin	Clone 14	1:30	BD Transduction Laboratories, Franklin Lakes, NJ

**Table 2:** Average of thickness and intensity of immunohistochemical staining signal for the different cellular proteins in each morphological class

	p16		MMP-7		MMP-16		CK8/18		Laminin		E-cadherin		b-catenin	
	th	int	th	int	th	int	th	int	th	int	th	int	th	int
Normal SE	0	0	1	1	1-2	1	0	0	0	0	1-2	2	1-2	2
Condyloma	0	0	1–2	1–2	2	1	0–1	2	0	0	2–3	2–3	2–3	2
CIN1	1–2	1–2	1–2	1–2	2–3	1–2	0–1	1–2	0–1	1	2–3	2–3	2–3	2–3
CIN2	1–2	2	1–2	1–2	2–3	1–2	0–1	1–2	0–1	1	2–3	2–3	2–3	2–3
CIN3	1–2	3	1–2	1–2	2–3	2–3	1	1	1–2	1	3	3	2–3	2–3
Normal CE	n.a.	0	n.a.	1	n.a.	1	n.a.	1-2	n.a.	0	n.a.	1	n.a.	1
AIS	3	3	0–1	1	1–2	1–2	0–1	1	0–1	1	3	3	2	2–3

Thickness (th) and intensity (int) of staining are evaluated in a scale from 0 (no staining) to 3 (full thickness of epithelium or very intense staining). Normal SE, normal squamous epithelium. Normal CE, normal columnar epithelium. b-catenin = beta-catenin. n.a., not applicable due to single layer nature of normal columnar epithelium.

## Results

In our previous work we showed that expression of the E5 oncogene of HPV type 16 leads to increased expression of E-cadherin and beta-catenin proteins in monolayer cell cultures and in three-dimensional collagen raft cultures [11]. We continued to perform immunohistochemical staining for these proteins in human cervical hrHPV-associated disease. Our material covered condyloma, CIN1-3 and AIS, as well as normal squamous and columnar epithelium. In each type of HPV associated disease, thickness and intensity of staining were separately graded 0–3. The averages for normal tissue and disease tissue are shown in Table 2.

In order to visualize the dysplastic area of cervical tissue, antibody to p16 (p16INK4a) was used as a surrogate marker for the presence of hrHPV. p16 is not expressed in normal squamous epithelium (Figure 1.1), condyloma tissue (Figure 1.2), or normal columnar epithelium (Figure 1.6). In hrHPV-associated dysplastic lesions (CIN1-3), cytoplasmic as well as nuclear staining for p16INK was seen, correlating with the severity of the lesion (Figure 1.3-5; Table 2). In CIN1, the lower part of the dysplastic epithelium was positive for p16INK, whereas the superficial koilocytotic areas remained negative. More intense staining covering an increasing proportion of the epithelial thickness was seen in CIN2 and CIN3. In AIS, staining for p16INK was strongly positive and shows clearly the dysplastic area of cervical epithelium (Figure 1.7). HPV associated neoplasia and carcinoma could thus be visualized by using p16 staining.

**Figure 1:**
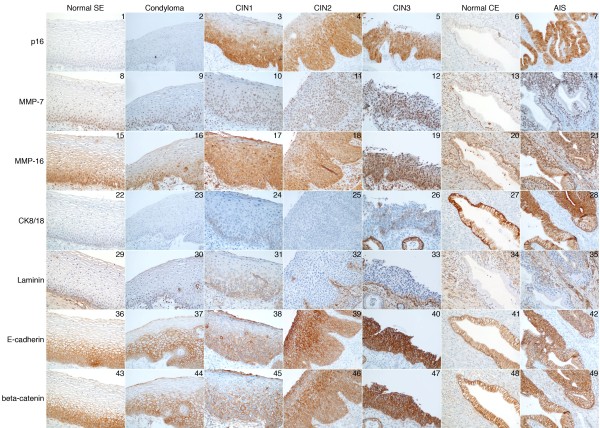
** Immunohistochemical staining for cellular proteins p16 (fields 1–7), MMP-7 (8–14), MMP-16 (15–21), cytokeratin 8/18 (CK8/18) (fields 22–28), laminin (29–35), E-cadherin (36–42) and beta-catenin (43–49) in different grades of high-risk HPV associated cervical disease.** SE, squamous epithelium; CE, columnar epithelium; AIS, adenocarcinoma *in situ*. Original magnification 20x.

Cervical tissue was stained for MMP-7 and MMP-16 (Figure 1.8-14, 1.15-21). Some background signal remained in MMP-16 staining despite modifications of the protocol. Both MMP-7 (Figure 1.8-9) and MMP-16 (Figure 1.15-16) are expressed in the nuclei of basal and suprabasal cells of normal squamous epithelium and condyloma. Mitotic figures are nicely stained, as well as inflammatory cells in the stroma. The expression of MMP-16 increases along with disease stage, and there is a strong tendency towards additional cytoplasmic staining for MMP-16 in the dysplastic area. The expression of MMP-7 remains altogether fairly similar in various disease stages (Figure 1.8-14; Table 2). Columnar cell nuclei and AIS were stained for MMP-7 and MMP16 as well, and a strong additional cytoplasmic staining was seen in AIS (Figure 1.14, 1.21). In preliminary experiments intense nuclear staining for MMP-12 was observed, but no grading along with disease status was seen (not shown). We previously found decreased MMP mRNA expression due to E5, however, the proteins levels remained unchanged [10].

The simple epithelial cytokeratin (CK) 8/18 showed no expression or very weak expression in basal/suprabasal cell cytoplasms along the basal lamina in normal squamous epithelium, condyloma or CIN1-2 (Figure 1.22-25). In CIN3, instead, individual cell cytoplasms were positive for CK 8/18 (Figure 1.26). These cells were scattered in all layers of the dysplastic tissue from basal cells up to the epithelial surface. The simple epithelial nature of CK 8/18 was emphasized by the strong basolateral staining in normal columnar epithelium (Figure 1.27). Intense cytoplasmic staining was observed in AIS (Figure 1.28).

Laminin staining was altogether relatively weak. A faint laminin staining was seen at the basement membrane of normal squamous epithelium and condyloma (Figure 1.29-30). In CIN1 a cytoplasmic staining starting from basal cells and reaching approximately one-third or one-half of the epithelium was observed (Figure 1.31). In CIN 2–3 a stronger staining in basal lamina but no cytoplasmic staining was seen (Figure 1.32-33). Some stromal staining was seen as well. Normal columnar epithelium was not stained for laminin, and in AIS occasional weak staining at the basolateral surface or in cell cytoplasms was observed (Figure 1.34-35). The laminin antibody recognizes laminins expressed at the basal lamina, but more accurate information about its exact reactivity to different laminin chains is not available. We also stained a subset of samples for fibronectin involved in cell adhesion, but the signal remained very weak altogether (not shown). The fibronectin antibody recognizes the extra domain A form of fibronectin [15].

Increased staining along with disease stage was found for the adherens junction proteins E-cadherin and beta-catenin. In normal squamous epithelium and condyloma membrane staining at adherens junctions covered approximately one-half of the epithelium (Figure 1.36-37, 1.43-44). In basal and suprabasal cells of normal epithelium cytoplasmic staining was observed as well. In CIN1, intense membrane staining covered two-thirds of the epithelial thickness, and in the differentiated cells of the surface the staining was weak or absent (Figure 1.38, 1.45). In CIN2, strong membrane staining covered the whole epithelium and occasionally a more intense zone was observed in the middle layers of the epithelium (Figure 1.39, 1.46). In CIN3, very strong membrane staining was seen throughout the dysplastic epithelium (Figure 1.40, 1.47). In CIN2-3 also cytoplasmic staining was seen in the dysplastic area. Faint basolateral staining for E-cadherin and beta-catenin was seen in normal columnar epithelium (Figure 1.41, 1.48). Strong membrane staining together with weak cytoplasmic reactivity for both proteins was observed in AIS (Figure 1.42, 1.49). In a subset of tissue samples stained for N-cadherin, a similar trend towards more intense staining along with the grade of lesion was seen (not shown).

**Figure 2:**
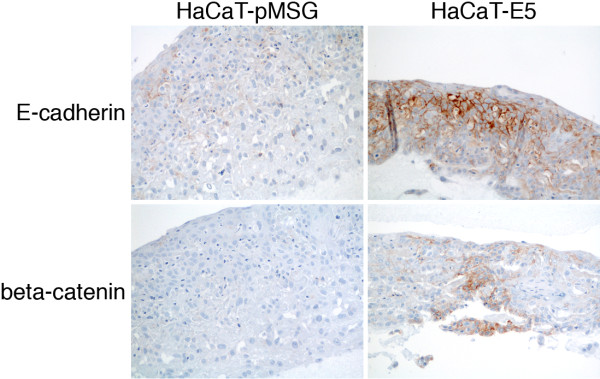
** Example of immunohistochemical staining in collagen raft cultures.** Staining for E-cadherin and beta-catenin in raft cultures established from human keratinocytes expressing HPV 16 E5 protein (HaCaT-E5) or control cells (HaCaT-pMSG). In each field the surface of the epithelial equivalent is facing upwards. Original magnification 20x.

Interestingly, in our previous work, stronger membrane staining for E-cadherin and beta-catenin was seen in E5 expressing three-dimensional epithelial raft cultures than in cells devoid of E5 [11]. Moreover, in that work, strongest staining was seen at and close to the surface of the epithelial structure, opposite to human cervical tissue. Figure 2 presents an example of E-cadherin and beta-catenin staining in raft cultures from HaCaT-E5 cells expressing HPV 16 E5 protein and HaCaT-pMSG control cells without HPV. Membrane staining for E-cadherin and beta-catenin was stronger in E5-expressing cells than in control cells. Both E5 and control raft cultures remained completely negative for p16, MMP-7, MMP-16, and CK8/18 (not shown). Raft cultures were not stained for laminin.

Altogether our results show the potential of experimental tissue culture settings to display the pathogenic events in human disease.

## Discussion

Here we have studied the expression of selected proteins implicated in cell adhesion, motility, and differentiation in cervical dysplasia associated with high-risk human papillomavirus infection. We have previously reported large-scale gene and microRNA profiling in epithelial cells expressing the HPV 16 E5 oncogene [10,11]. In those studies the E5 protein was found to affect crucial cellular pathways including cell adhesion, cell motility, extracellular matrix and mitogenic signaling, and those effects were partially shown to be mediated by microRNA regulation [10,11].

In the present work we sought out to study whether altered cellular gene expression profiles found in an experimental setting would apply to human cervical disease. We studied altogether 50 samples representing human cervical disease of different grades, together with four samples representing normal squamous epithelium and two from columnar cervical epithelium. The aim of the study was to perform preliminary biological validation of gene expression microarray findings in human disease, and to search for putative feasible biomarkers for disease diagnostics. The study was limited by the availability of reliable antibodies to cellular proteins for immunohistochemical staining. Further, the sample number per histological grade is rather small and thus our results are rather of suggestive nature. However, we were able, by using tissue samples from human cervical disease, to validate trends of altered expression of several genes which were first identified in microarray.

Matrix metalloproteinases are proteins that degrade the extracellular matrix and are thus essential in a number of physiological processes and pathological conditions including embryonic development, differentiation, tissue remodeling and cancer [12], also in cervical cancer [16]. Specifically the involvement of MMP-2, MMP-9 and MMP-14 in cervical carcinogenesis has been reported [17-19]. Expression of MMP-7 has been found to correlate with invasive property and advanced tumor stage of head and neck squamous cell carcinoma [20], and endometrial [21] and gastric adenocarcinoma [22]. This seems to be tumor type specific, however, since most cervical neoplasia and carcinoma cases were found negative for MMP-7 [17]. Interestingly, in a previous study no expression of MMP-12 or MMP-16 was found in HPV harboring cervical carcinoma cell lines [23].

In the present study we found increasing staining for MMP-16 along with the development of cervical dysplasia, in agreement with a number of previous studies, whereas MMP-7 expression remained virtually similar. Although the individual role of the E5 oncogene in the carcinogenic process is not fully understood, this seems to disagree with our previous finding of reduced MMP-7 and MMP-16 mRNA expression in monolayer cells expressing the E5 protein [10], albeit protein expression in that study remained unchanged, similar to what we have shown here for MMP-7 in tissue samples. That result may have been due to increased half-life of the transcripts or increased degradation of the protein. Altogether, effects of the E5 oncogene as seen in microarray may only represent early and/or partial consequences of human papillomavirus in infected epithelial cells. In this work, both MMP-7 and MMP-16 were found to be expressed in the nuclei of basal and suprabasal cells of normal squamous epithelium and condyloma. Most reports describe MMP localization at the level of tissue, not within cells, and only a few papers discuss the function of nuclear MMP localization. However, nuclear localization of proteolytically active MMP-2 in cardiac myocytes, and MMP-3 in cultured cells and in human liver tissue has been shown [24,25]. MMP-2 cleaves PARP in the nucleus of cardiac myocytes, and the authors speculate that this might inhibit DNA damage repair by PARP [24]. The effects of PARP inhibition in cancer, however, have been shown to be contradictory [26]. Nuclear MMP-3 seems to be involved in the induction of apoptosis [25]. Although our findings do not allow further speculation due to modest changes in MMP expression, the putative functions of nuclear MMP’s are worth further studies considering the process of cervical carcinogenesis.

Keratin-8 is often paired with keratin-18 and is expressed in simple epithelia [27]. Here we show reasonably strong scattered expression of cytokeratin-8/18 in severe intraepithelial neoplasia. The expression of CK8/18 in squamous epithelium is an unresolved issue, although its expression has been previously shown in high-grade CIN and squamous cell cervical cancer [27-29]. Poor prognosis has been correlated with cytokeratin 8/18 expression in squamous cell carcinoma of the oral cavity [30]. CK8/18 expression was shown in carcinoma induced by inoculating nude mice with tumorigenic HPV 16-immortalized keratinocytes [31]. Our results agree with the previous data, because normal squamous epithelium, low-grade lesions, and even CIN2 were basically negative for CK8/18, whereas strong although scattered expression was shown in CIN3.

Laminin-5 enhances cellular migration and tumorigenicity, and it has been shown to be overexpressed in cervical cancer [32]. High-risk HPV E6 oncogene has been previously shown to upregulate laminin-5 receptor expression in cervical cancer cells [33]. This takes place by E6-mediated downregulation of human miR-218, leading to upregulation of its target gene LAMB3, which is a component of the laminin-5 receptor expressed in the basal lamina [33]. On the contrary, in our study the staining for laminin was strongest in CIN1, whereas in other tissues laminin was seen only in the basal lamina. Unfortunately exact information about the reactivity of the antibody was unavailable and thus we cannot further speculate about the role of laminin in CIN1. This is, however, an extremely interesting finding, considering the possible early events towards carcinogenesis being evident already in a low-grade lesion.

Carcinogenesis essentially involves downregulation of E-cadherin and disruption of E-cadherin–beta-catenin complexes in adherens junctions [9]. Along with the malignant development, the E-cadherin–beta-catenin complex at the plasma membrane is degraded and beta-catenin is transported to the nucleus where it acts as a transcription factor in the Wnt signaling pathway and contributes to malignant development. Differences in intercellular junctions even among morphologically similar cells may be a manifestation of a crucial event in cervical carcinogenesis [34,35]. The expression of E-cadherin and beta-catenin in HPV-associated cervical neoplasia is, however, equivocal. Decreased and cytoplasmic expression of those proteins in cervical cancer has been reported [36-39]. Leong et al. showed weakening and dysregulated E-cadherin expression in HPV harboring lesions [40]. Those authors showed that although downregulation of E-cadherin has previously been associated with the development of neoplasia and cancer, it seems to have little to do with the carcinogenic process and seems to be unrelated to the ability of the E6 and E7 proteins to bind and degrade pRb or p53 [40]. Quite the opposite, in the present work we found increased membrane and cytoplasmic staining for both E-cadherin and beta-catenin in high-grade cervical lesions. In agreement with this, Samir et al. reported increased E-cadherin expression with increasing CIN grade [41].

We have previously shown increased expression of E-cadherin and beta-catenin due to E5 expression in epithelial cells [11]. We further observed downregulation of cellular miR-324-5p, which we showed to putatively target both E-cadherin and beta-catenin, and thus its downregulation would lead to increased expression of the target proteins [11]. In the present work, in agreement with previous observations, we observed increased expression of E-cadherin and beta-catenin due to E5, E6 and E7 oncogenes in three-dimensional collagen raft cultures. This is in agreement with the increased expression found in high-grade human cervical tissue. Interestingly, most intense staining in E5-expressing raft cultures was seen in the most differentiated layers of the raft epithelium, contrary to normal tissue, condyloma or CIN1, where most intense staining was seen in the bottom layers of the epithelium. In raft cultures expressing the E6 and E7 oncogenes the strongest staining was seen in the lower part of the raft epithelium, similar to human tissue. This repeated observation might be explained by decreased expression of adhesion molecules towards fully differentiated and desquamating cells which are found in normally maturing but not in neoplastic epithelium.

Extracellular matrix-degrading proteins may be involved in modification of intercellular junctions. It has been speculated that MMP-7 expressed on the surface of carcinoma cells may cleave E-cadherin and facilitate the detachment of carcinoma cells from the site of primary tumor [42], although we did not see this inverse relationship of expression in our work. The expression of the secreted extracellular matrix-degrading proteinase MMP-7 has been shown to be upregulated by beta-catenin in colon tumor cell lines, and a crucial role prior to the onset of the invasive phenotype has been suggested [43]. We observed increased membrane and cytoplasmic staining for beta-catenin along with the severity of the lesions, including AIS, whereas MMP-7 expression remained virtually similar. Regulation of various cellular functions participating in carcinogenesis is complex. For example, the role of integration of the HPV genome into the chromosome in the differential expression of cellular proteins is poorly understood.

For normal epithelial development, basal epithelial cells have to detach from integrin and laminins of the basement membrane, cease to proliferate, and enter the differentiation program. In epithelial dysplasia or cancer, cells do not differentiate but continue to proliferate. Many of the phenomena seen in cervical intraepithelial neoplasia and cancer are the results of viral oncoprotein functions. A number of observations based on cervical morphology and tissue staining have led to better understanding of cervical pathogenesis. Also, careful examination of viral functions in experimental systems contribute to our knowledge of carcinogenesis and, importantly, may provide novel biomarkers for the diagnosis, follow-up and cure of cervical disease.

## Conclusion

In conclusion, our results suggest that experimental approaches such as tissue culture and microarray can provide valuable preliminary insight into the pathogenesis of human disease. Papillomavirus biology and the effects of HPV in infected cells are not fully understood. Importantly, optimally prognostic biomarkers would be of great impact in the management of HPV associated disease, where only a minor but clinically important subset finally develops into severe lesions and cancer.

## Abbreviations

AIS: adenocarcinoma *in situ*; CIN: cervical intraepithelial neoplasia; CK: cytokeratin; EGFR: epidermal growth factor receptor; HPV: human papillomavirus; hrHPV: high-risk human papillomavirus; MMP: matrix metalloproteinase; PARP: poly(ADP-ribose) polymerase.

## Competing interests

The authors have no competing interests to declare.

## Authors’ contributions

NK performed most experiments, collected the data, analyzed and interpreted the results and participated in drafting the manuscript. MR collected the data, analyzed and interpreted the results and participated in finalizing the manuscript. JT collected the sample material, analyzed and interpreted the results and participated in finalizing the manuscript. PA participated in the study design and in finalizing the manuscript. EA performed main design of the study, colllected the data and prepared the manuscript. All authors read and approved the final manuscript.

## Pre-publication history

The pre-publication history for this paper can be accessed here:

http://www.biomedcentral.com/1472-6890/12/9/prepub
